# Altered functional connectivity between hypothalamus and limbic system in fibromyalgia

**DOI:** 10.1186/s13041-020-00705-2

**Published:** 2021-01-20

**Authors:** Jian Kong, Yiting Huang, Jiao Liu, Siyi Yu, Cheng Ming, Helen Chen, Georgia Wilson, William F. Harvey, Wen Li, Chenchen Wang

**Affiliations:** 1 Department of Psychiatry, Massachusetts General Hospital, Harvard Medical School, Charlestown, MA 02129 USA; 2 Center for Complementary and Integrative Medicine, Division of Rheumatology, Tufts Medical Center / Tufts University School of Medicine, Boston, MA 02111 USA; 3 Department of Psychology, Florida State University, Tallahassee, FL USA

**Keywords:** Resting state functional connectivity, Fibromyalgia, Limbic system, Hypothalamus, Diencephalon, Mind–body intervention

## Abstract

The hypothalamus links the nervous system to the endocrine system and plays a crucial role in maintaining the human body's homeostasis. This study aims to investigate the resting state functional connectivity (rsFC) changes of the hypothalamus in fibromyalgia patients. 24 Fibromyalgia patients and 24 matched healthy controls (HCs) were recruited. Resting state fMRI data were collected from the fibromyalgia patients and HC’s. Fibromyalgia patients went through a second scan after 12 weeks of Tai Chi mind–body intervention. Data analysis showed that fibromyalgia patients displayed *less* medial hypothalamus (MH) rsFC with the thalamus and amygdala when compared to the functional connectivity in the HCs. After the Tai Chi mind–body intervention, fibromyalgia patients showed *increased* MH rsFC with the thalamus and amygdala accompanied by clinical improvement. Effective connectivity analysis showed disrupted MH and thalamus interaction in the fibromyalgia patients, which was altered by mind–body exercise. Our findings suggest that fibromyalgia is associated with altered functional connectivity within the diencephalon and limbic system. Elucidating the roles of the diencephalon and limbic system in the pathophysiology and development of fibromyalgia may facilitate the development of a new biomarker and effective treatment methods for this prevalent disorder.

*Trial Registration* ClinicalTrials.gov, NCT02407665. Registered: 3 April 2015, https://clinicaltrials.gov/ct2/show/NCT02407665?term=NCT02407665&draw=2&rank=1

## Introduction

Fibromyalgia is a complex disorder characterized by chronic and widespread musculoskeletal pain [1]. Although still under investigation, accumulating evidence has suggested that the central nervous system plays a pivotal role in the pathophysiology of fibromyalgia. As a result, brain imaging tools have been utilized extensively to investigate the pathophysiology [2–11], treatment response [12–15] and classification [16] of fibromyalgia.

Accumulating evidence suggest that the reorganization of the limbic system may play an important role in chronic pain [17–19]. As a center of the limbic system, the hypothalamus links the endocrine and nervous systems, maintains the body’s internal balance (homeostasis), and regulates vital bodily functions such as stress, immune responses, and autonomic and endocrine functions [20, 21]. Literature suggests that hypothalamic subregions are anatomically connected to the frontal lobes, hippocampus, thalamus, and the brain stem for the integration of sensory and affective information; all of these regions play a crucial role in chronic pain and its regulation [19, 22, 23].

In addition, as the initial point of the hypothalamic–pituitary–adrenal axis activation, the hypothalamus has been found to exert regulatory effects on the inflammatory and stress response [20, 24], which may play an important role in the pathophysiology of chronic pain, including fibromyalgia [25, 26]. Brain imaging studies suggest that it may also be involved in chronic pain. For example, studies have found activation within the hypothalamic region during spontaneous bouts of chronic pain [27]. Nevertheless, the role of the extended hypothalamus system in the pathophysiology and development of fibromyalgia remains unclear.

Recently, resting state functional connectivity (rsFC) has been widely used in pain research. Investigators have found that brain oscillations and synchrony, which can be measured by rsFC, can provide information about the intrinsic functional organization of the brain [28, 29] and may play a crucial role in the information flow of pain processing and modulation [23, 30–34].

Thus, the aim of this study is to investigate the role of hypothalamus rsFC in the pathology as well as the development of fibromyalgia. Specifically, we first investigated the hypothalamus (medial and lateral separately) rsFC difference between the patients and healthy controls, then explored how the hypothalamus rsFC changes following symptom reductions after non-pharmacological Tai Chi treatment. We chose Tai Chi mind–body intervention because our previous study demonstrated that Tai Chi can significantly improve clinical outcomes in patients with fibromyalgia [35, 36]. We hypothesized that (1) fibromyalgia patients will be associated with altered hypothalamus rsFC with the limbic system, and (2) some altered hypothalamus functional connectivity changes, as detected above, will normalize (similar to the functional connectivity in the healthy controls) after mind–body intervention in patients with fibromyalgia.

We believe our experimental design will help us deepen our understanding of the central mechanism of fibromyalgia. For instance, one area that remains unclear is if the brain regions that showed differences between fibromyalgia patients and healthy control subjects (HC’s) are also sensitive to symptom reduction after treatments. Theoretically, brain regions that show functional differences between fibromyalgia patients and HC’s may be used as potential biomarkers for distinguishing fibromyalgia patients from HC’s. Additionally, brain regions that are sensitive to pain intensity changes may have potential for fibromyalgia severity monitoring and may act as an objective measurement for treatment response [37].

## Results

Five healthy controls were excluded (one had brain atrophy and four reported some level of pain in the past week). Three patients were dropped from the study due to schedule conflicts, and did not participate in the second MRI scan. One fibromyalgia patient was excluded from rsFC analysis due to excessive head movement during the scan. Final analysis was performed on 20 fibromyalgia patients and 19 pain-free controls.

The mean age of the fibromyalgia subjects (n = 20) was 51.6 ± 11.6 (mean ± SD) and was 52.3 ± 10.4 for the control subjects (n = 19). There were no significant differences in age and gender between the fibromyalgia and healthy control groups (see Additional file 1: Table S1 for data of each individuals).

FIQR scores demonstrated moderate to severe fibromyalgia in the majority of fibromyalgia subjects with an average score of 45.9 ± 17.6 (mean ± SD). BDI-II scores revealed moderate depression with an average score of 17.7 ± 9.3 in the fibromyalgia group, and a two-sample t-test showed significant differences (p < 0.001) between the fibromyalgia and healthy control groups in BDI-II scores (4.2 ± 3.2). Paired t-tests showed significant pre- and post-Tai Chi differences in general FIQR scores (Pre: 45.9 ± 17.6, post: 36.3 ± 20.3, p = 0.001) and three sub scores: Function (p < 0.001), Overall Impact (p = 0.03), and Symptoms, (p = 0.02). Analysis of BDI-II scores demonstrated a significant difference between pre- and post-treatment scores in fibromyalgia patients (pre: 17.7 ± 9.3, post: 10.8 ± 9.2, p < 0.001). There was also a significant association between FIQR score changes and BDI score changes (p = 0.003). Please see Additional file 1: Table S1 for clinical outcomes of individuals who were included in data analysis.

### Medial hypothalamus (MH) rsFC results

**Fig. 1:**
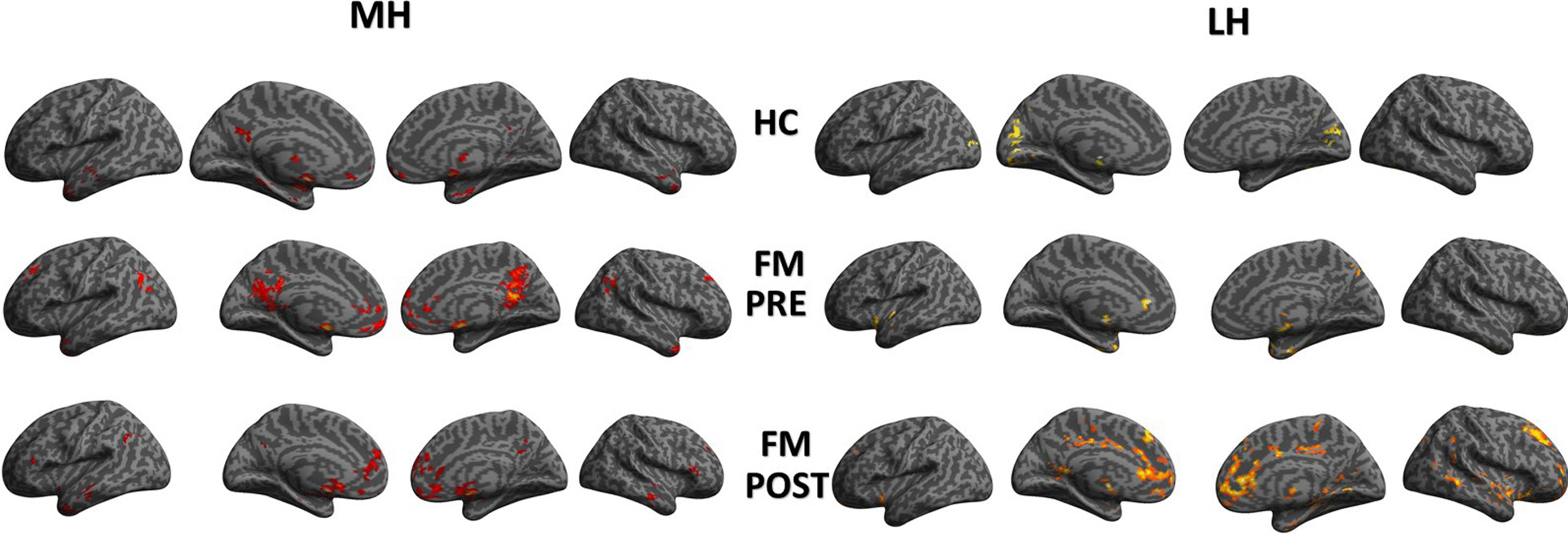
Resting state functional connectivity of the medial hypothalamus (MH) and the lateral hypothalamus (LH) in fibromyalgia patients and healthy controls. A threshold of p < 0.005 with 80 continuous voxels was applied

The MH rsFCs of the fibromyalgia patients (pre- and post-intervention) and HCs are presented in Fig. 1. The results showed that both fibromyalgia and HC are associated with positive MH rsFC in brain regions that belong to the default mode network (medial prefrontal cortex (MPFC)/anterior cingulate cortex (ACC), posterior cingulate cortex (PCC)/precuneus) and bilateral thalamus. Of the three conditions, fibromyalgia (pre-treatment) was associated with the most robust positive MH rsFC.

Compared to the healthy controls, fibromyalgia patients at baseline showed significantly increased rsFC between the medial hypothalamus and bilateral subcallosal cingulate cortex and showed decreased rsFC between the medial hypothalamus and bilateral cerebellum, amygdala (k = 2), and right thalamus (k = 5) at baseline (Table 1).

**Table 1:** Regions showing significantly different functional connectivity with the medial hypothalamus (MH) and lateral hypothalamus (LH) in fibromyalgia patients (FM) and healthy controls, before and after three-month intervention

Seed	Condition	Region	Cluster size	MNI coordinates			Peak z value
				X	Y	Z	
MH	FM (pre) > HC	Bilateral subcallosal cingulate gyrus	125	− 12	14	− 16	3.85
	HC > FM (pre)	Bilateral cerebellum	155	0	− 80	− 28	3.82
		Left amygdala	70	− 30	− 8	− 16	4.72
		Right amygdala	23	32	− 8	− 20	3.12
		Right thalamus	32	10	− 14	0	3.54
	FM (post) > FM (pre)	Right DLPFC/operculum	105	56	20	2	4.16
		Right occipital gyrus	180	22	− 78	− 6	4.23
		Left cerebellum	101	− 12	− 70	− 20	3.96
		Left amygdala	24	− 18	− 12	− 12	3.64
		Right thalamus	59	8	− 18	2	4.19
		Left rACC	23	− 12	44	22	3.8
	FM (pre) > FM (post)	None					
LH	FM (pre) > HC	Right temporal pole	152	38	6	− 42	3.54
	HC > FM (pre)	Right inferior occipital gyrus/cerebellum	213	40	− 66	− 8	3.93
	FM (post) > FM (pre)	Bilateral rACC	229	10	36	6	4.42
		Bilateral MPFC	180	8	46	− 2	4.1
		Right middle temporal gyrus/superior temporal gyrus	185	60	0	− 16	4.34
		Left PCC	106	− 12	− 36	34	4.09
	FM (pre) > FM (post)	None					

Cluster size indicates the number of voxels in the cluster; the size of each voxel is 8 mm^3^

*FM (pre)* fibromyalgia pre-Tai Chi intervention, *FM (post) *fibromyalgia post-Tai Chi intervention, *HC* healthy control, *DLPFC *dorsolateral prefrontal cortex, *PAG* periaqueductal gray, *rACC* rostral anterior cingulate cortex, *MPFC* medial prefrontal cortex, *PCC* posterior cingulate cortex

A direct comparison of before and after mind–body intervention in fibromyalgia patients indicated an increased medial hypothalamus rsFC within the left amygdala (k = 2), rACC (k = 5), cerebellum, right DLPFC/operculum, thalamus (k = 5), and occipital area after Tai Chi intervention. No reduced MH rsFC was found after Tai Chi intervention at the threshold we set (Table 1).

Interestingly, we found that increased MH-thalamus and MH-amygdala rsFC after mind–body intervention overlapped with decreased MH-thalamus rsFC when compared with the healthy control group, indicating that the treatment can normalize decreased MH FC with thalamus and amygdala.

**Fig. 2:**
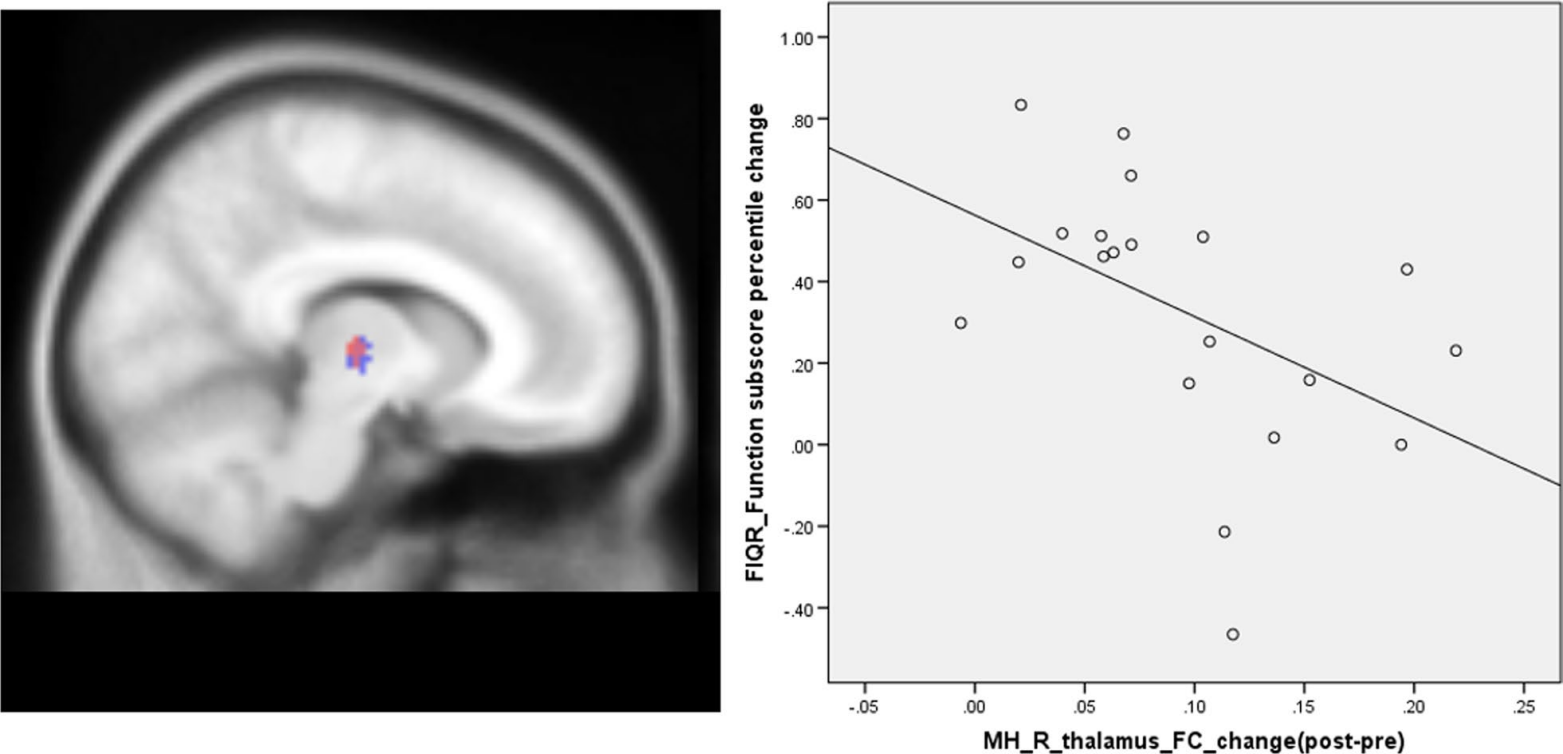
Medial hypothalamus functional connectivity decreased in the right thalamus in FM patients compared to healthy controls (red) and increased after 12-weeks of Tai Chi intervention (blue). The scatter plot indicates the significant association between the MH-thalamus functional connectivity changes and the FIQR function sub-score percentile changes

Exploratory analysis between the FIQR and subscores with the MH-thalamus connectivity showed a significant correlation between MH-thalamus connectivity changes and FIQR function sub-score percent changes before and after mind–body intervention (r = − 0.48, p = 0.03, uncorrected for multiple comparison) (Fig. 2). There are no other significant associations between the MH-thalamus connectivity change and the clinical outcome changes (FIQR and subscores percent changes).

### Lateral hypothalamus (LH) rsFC results

LH rsFC of the fibromyalgia patients and HC’s is presented in Fig. 1. Results showed that HC’s were associated with positive LH rsFC within the thalamus and occipital cortex, while fibromyalgia was associated with positive LH rsFC within the insula and brain regions belonging to the default mode network, including the ACC/MPFC and precuneus. This pattern was similar after Tai Chi intervention, but the connectivity was more robust.

Compared to the HCs, fibromyalgia patients (at baseline) showed significantly greater rsFC between the LH and the right temporal pole and decreased rsFC between the lateral hypothalamus and the right occipital inferior gyrus/cerebellum (Table 1).

Comparisons before and after Tai Chi intervention in fibromyalgia patients indicate an increased rsFC between the lateral hypothalamus, the bilateral rostral anterior cingulate cortex (rACC), and the MPFC, as well as the left PCC and right middle temporal gyrus/ superior temporal gyrus. There was no significant rsFC reduction after Tai Chi intervention (Table 1).

Exploratory analysis between the FIQR and subscores with the LH-thalamus connectivity showed no significant association between the MH-thalamus connectivity change and the clinical outcomes changes (FIQR and subscores percent changes).

### Effective connectivity of medial hypothalamus (MH)

**Fig. 3:**
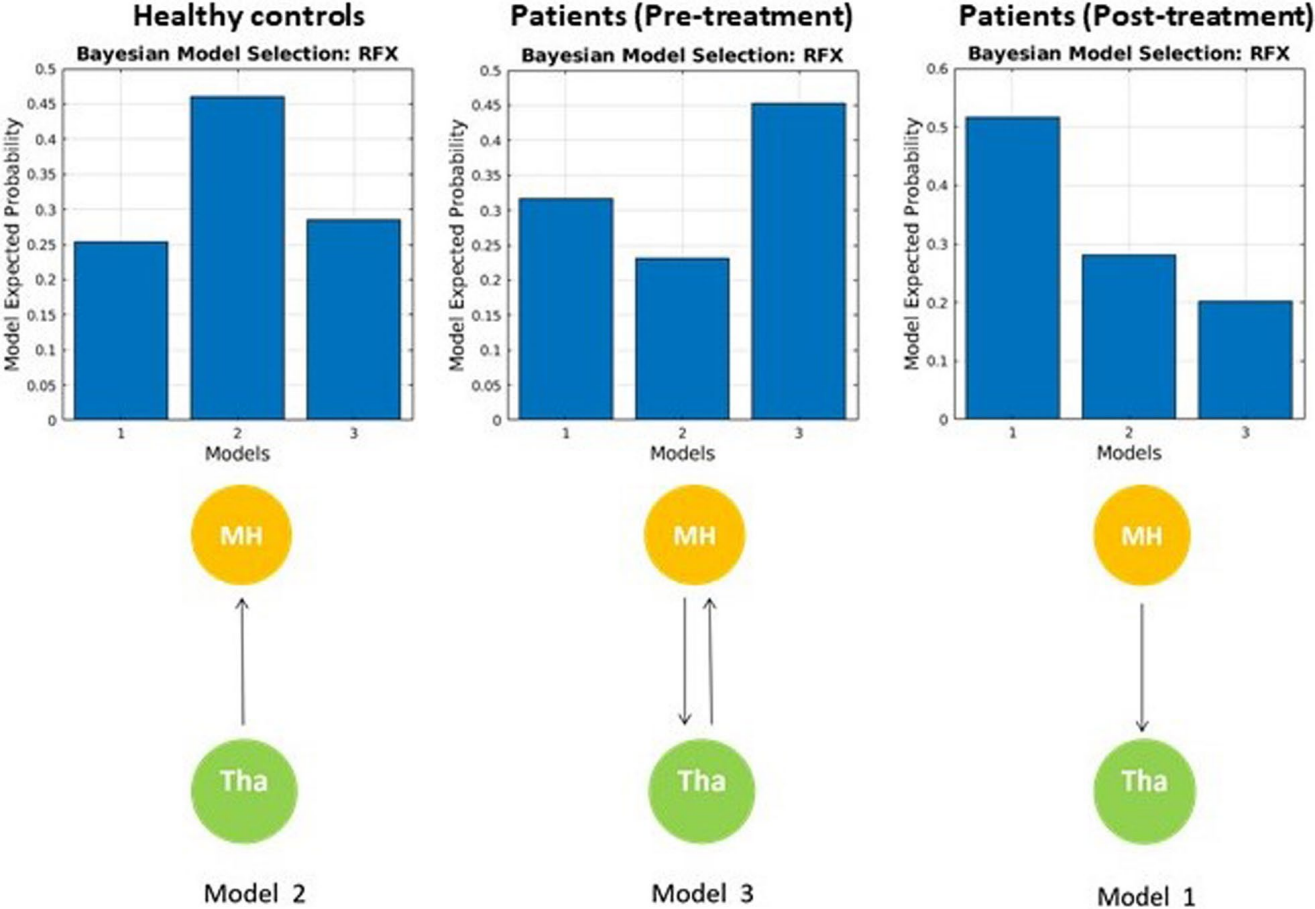
Medial hypothalamus–thalamus effective connectivity model and results

The results of effective connectivity analysis at the group level are shown in Fig. 3. For healthy subjects, the best model at the group level was Model 2. For patients with fibromyalgia, Model 3 was the best for pre-intervention, while Model 1 was the best for post-intervention.

## Discussion

In this study, we investigated medial and lateral hypothalamus functional connectivity in fibromyalgia patients and connectivity changes following a mind–body intervention. We found that (1) compared to healthy controls, fibromyalgia patients were associated with less MH rsFC with the thalamus and amygdala; (2) the MH rsFC with the thalamus and amygdala increased (normalized), and the MH-thalamus rsFC was significantly associated with clinical outcome changes after the Tai Chi mind–body exercise; and (3) there was an MH/LH rsFC increase with the rACC after intervention. In addition, we found different MH-thalamus effective connectivity between the fibromyalgia patients and HC’s, as well as before and after mind–body treatment. Our results suggest that fibromyalgia is associated with altered functional connectivity within the limbic system, and some of these changes may be sensitive to mind–body intervention.

The hypothalamus is a small but functionally diverse region of the brain, exerting vital regulatory influences over the central and peripheral nervous system. As a key region of the limbic system, it has direct connections with the frontal lobes, hippocampus, amygdala, thalamus, and brain stem, and regulates many autonomic processes. In this study, we found two overlapping but distinct neural networks from the LH and MH using resting-state FC analyses. Our results are consistent with the findings from a previous study on healthy subjects using an identical seeds as the current study [21].

One major finding of our study is the decreased MH-thalamus rsFC in fibromyalgia patients compared with the rsFC in the HC’s and the increased MH-thalamus rsFC after Tai Chi mind–body intervention. Both the hypothalamus and thalamus belong to the diencephalon, and there are bidirectional connections between these two structures. The thalamus is a key region for central processing and integration of nociceptive inputs. It acts as a relay center for handling incoming sensory information and motor impulses between the spinal cord, medulla oblongata, and cerebrum. Specifically, the thalamus receives nociceptive signals via two major ascending pathways: the spinothalamic tract (STT) and the spinoreticulothalamic tract (SRT). The STT conveys noxious information from the dorsal horn to both the lateral thalamus and medial thalamus, while the SRT mainly relays nociceptive information to the medial thalamus via an additional synaptic relay within the medullary reticular formation of the brainstem [38].

Further studies suggest that the thalamo-cortical pathways/interactions may underlie the perception of pain as an unpleasant sensory and emotional experience. The lateral thalamocortical pathway is involved in coding the sensory discriminative aspects of pain, while the medial thalamocortical pathway codes the emotional qualities of pain [38]. Literature suggests that the anatomical and biochemical alterations in thalamocortical circuits may be responsible for the development of chronic pain [33, 38–40]. The thalamus observed in our study extends from the medial portion to the lateral portion of thalamus, suggesting alterations of both sensory and emotional aspects in the pathophysiology of fibromyalgia.

We also found that fibromyalgia patients are associated with less MH rsFC with the amygdala. After effective mind–body intervention, the MH rsFC in the amygdala significantly increased. The amygdala is a key region in the limbic system that plays an important role in emotion processing, fear and anxiety response, and the influence of negative emotions on pain [41]. The amygdala is also part of the descending pain modulation system, directly projecting to the PAG [42]. A previous study showed that chronic low back pain-evoked brain activity increases in the amygdala and rACC/MPFC [43] and is associated with volume decreases in the amygdala [44]. A more recent study found higher incidences of white matter and functional connections within the MPFC–amygdala–accumbens circuit, with smaller amygdala volume accounting for 60% of the variance for chronic low back pain persistence [45]. Our results agree with these findings, suggesting that the linkage between the MH and amygdala may play an important role in the pathophysiology and development of fibromyalgia.

After mind–body intervention, both MH-rACC and LH-rACC rsFC increased, accompanied by clinical improvement. This result is consistent with a previous study using the same data set, in which we found that DLPFC rsFC with the rACC significantly increased after Tai Chi [14]. Similar results have been observed after various exercise interventions (Tai Chi, Baduanjin, and stationary cycling) in patients with knee osteoarthritis and after transcutaneous vagus nerve stimulation in patients with depression [46]. As a key region of the limbic system, default mode network, and descending pain modulation system, the role of the rACC in the pathophysiology of chronic pain has been well-documented [5, 37, 47–51]. Previous studies have suggested close functional connectivity between the PAG, a key region of the descending pain modulation system, and the rACC [52], and alterations in PAG-rACC functional connectivity in patients with fibromyalgia [5].

Literature suggests that the hypothalamus has direct ascending and descending connections with the PAG [27]. In this study, we did not observe the hypothalamus rsFC changing with the periaqueductal gray at the threshold we set. Nevertheless, at a less conservative threshold of p < 0.005 (voxel-wise uncorrected), we did detect an MH-PAG rsFC decrease (peak MNI coordinate 4, − 28, − 8, 8 voxels) in fibromyalgia patients when compared to HC’s and an MH-PAG rsFC increase after intervention (peak MNI coordinate − 2, − 24, − 12, 12 voxels). Taken together, we speculate that Tai Chi intervention may work by modulating the linkages between key regions of the limbic system, including the hypothalamus, rACC, amygdala, and thalamus, as well as the functional connectivity of key regions between the limbic and descending pain modulation systems such as the PAG. It is worth noting that the rACC and amygdala are key regions of both the limbic system and descending pain modulation system [42, 52, 53], which provide further support for our hypothesis.

Our findings of clinical improvement after 12-weeks of Tai Chi exercise are consistent with a previous study demonstrating the positive effects of Tai Chi [36] and exercise [12] in fibromyalgia patients. In addition, our results are consistent with previous brain imaging studies, which demonstrated a significant modulation effect of Tai Chi on brain function and structure in healthy human subjects [54–61] and chronic pain patients [62, 63]. Nevertheless, the lack of a control condition has significantly limited our ability to identify the precise mechanism behind Tai Chi’s therapeutic effects. We would like to emphasize here that the aim of this study was NOT to investigate the mechanism of Tai Chi, but to investigate the rsFC alterations of the hypothalamus in fibromyalgia patients and how these rsFC’s changed following effective treatment with symptom relief. We believe this study may shed light on our understanding of the pathophysiology and development of fibromyalgia.

In this study, three of 20 fibromyalgia patients maintained their regular pharmacological medications in addition to Tai Chi. Thus, confounding factors affecting association of pain medications, placebo effect (interaction between the Tai Chi instructors and patients), regression to the mean, and other unknown factors may also have contributed to the clinical improvements observed but this is beyond the scope of this manuscript. Moreover, we did not track the details of the medications that the participants were taking during and after treatment, and this should also be considered a limitation of the study.

Finally, DCM analyses indicated different MH–thalamus connectivity patterns between the fibromyalgia and healthy control groups, as well as before and after Tai Chi mind–body intervention with clinical symptom reduction. In healthy subjects, the effective connectivity analysis showed that the thalamus influences the MH (Model 2). In contrast, in fibromyalgia patients, the effective connectivity was bidirectional at the pre-treatment, including the opposite direction of the MH driving the thalamus (Model 3). After mind-body intervention, fibromyalgia patients exhibited a major change in the effective connectivity, represented by unidirectional connectivity with the MH driving the thalamus (Model 1). These results suggested that fibromyalgia is associated with significant anomalies in the MH-thalamus interaction. Interestingly, mind-body Tai Chi intervention did not appear to normalize the interaction, but rather demonstrated the opposite effective connectivity (MH-to-thalamus connectivity) compared to that of the control group. We speculate this may implicate that Tai Chi can first influence the activity of the hypothalamus by regulating stress, anxiety, and depression in patients with fibromyalgia, which can further influence the sensory (pain) perception/processing at the thalamus, to achieve clincial improvement.

In conclusion, we found that compared to healthy controls, fibromyalgia patients showed altered functional connectivity within the limbic system and diencephalon (hypothalamus, thalamus, amygdala, and ACC). After the Tai Chi mind–body intervention, decreased MH-thalamus/amygdala rsFC increased. Effective connectivity analysis showed that the MH and thalamus interaction was disrupted in fibromyalgia patients, and Tai Chi intervention could alter this interaction to achieve clinical improvement. Elucidating the role of the extended hypothalamus network in fibromyalgia may shed light on the pathophysiology and development of this prevalent disorder and facilitate the development of a new treatment for chronic pain.

## Materials and methods

This study was registered on ClinicalTrials.gov (NCT02407665). The protocol was applied by Tufts Medical Center/Tufts University Human Institutional Review Board and the Medical Ethics Committee of Massachusetts General Hospital. The full details of the study are reported in a previous study, in which we investigated dorsal lateral prefrontal cortex (DLPFC) rsFC differences between fibromyalgia patients and healthy controls and the modulation effect of Tai Chi exercise [14]. In this study, we investigated the rsFC of the lateral and medial hypothalamus between the fibromyalgia patients and healthy controls and the modulation effect of Tai Chi on hypothalamus connectivity, which has not been previously published.

### Participants

Based on the American College of Rheumatology (ACR) 1990 classification criteria and the ACR 2010 diagnostic criteria for fibromyalgia, 24 patients (≥ 21 years old) with fibromyalgia and 24 pain-free healthy controls matched for age, gender, and body mass index (BMI) participated in the study. The main exclusion criteria were: (1) diagnosis of medical conditions that are known to contribute to fibromyalgia symptomatology, (2) inability to pass the Physical Activity Readiness Questionnaire, (3) a score of less than 24 on the Mini-Mental State Examination; (4) if the patient presented any contraindications to fMRI scanning or had prior experience with Tai Chi training; and 5) similar types of complementary and alternative medicine in the past year.

### Intervention

All participants in the Tai Chi group attended a 60-min practice session twice a week for 12 weeks at Tufts Medical Center using a standardized Tai Chi protocol developed for patients with fibromyalgia [36]. Each component of the program was derived from the condensed version of the classical Yang-style, 108-posture Tai Chi for the 12-week intervention program. Participants were also instructed to practice at least 30 min a day at home. All subjects were encouraged to maintain their usual physical activities and to perform no new additional strength training other than their Tai Chi exercises. Subjects were also allowed to continue taking their regular medications and maintain routine physician visits throughout the course of the study. We did not track the medication use during the Tai Chi intervention and at the end of the study.

### Outcome measurements

The primary outcome for this study was the resting state functional connectivity (rsFC) of the medial and lateral hypothalamus. Secondary outcomes included: (1) Revised Fibromyalgia Impact Questionnaire (FIQR) including the three domains, i.e., Function, Overall Impact, and Symptom Severity, and (2) Beck Depression Inventory (BDI-II). All outcome measurements were collected at baseline for all subjects and after 12 weeks of the intervention for the fibromyalgia patients.

### MRI data acquisition

fMRI scans were performed at the Martinos Center for Biomedical Imaging of Massachusetts General Hospital with a 32-channel head coil and 3.0 T Siemens (Skyra syngo) scanner. Magnetization-Prepared Rapid Gradient Echo (MPRAGE) T1-weighted images were collected (voxel size 1.0 × 1.0 × 1.0 mm^3^). The blood oxygen level dependent (BOLD) resting state functional images were obtained with echo-planar imaging (TR = 3000 ms, TE = 30 ms, flip angle = 85°, slice thickness = 2.6 mm, acquisition matrix = 64 × 64, voxel size = 2.6 × 2.6 × 2.6 mm^3^, 44 axial slices, scan time 8 min 21 s). All patients were required to keep their eyes open during the resting state fMRI scan.

### Statistical analysis

#### Clinical data analysis

Clinical data analysis was performed using SPSS 19.0 software (SPSS Inc., Chicago, IL, USA). A threshold of p < 0.05 (2-tailed) was applied. T-test and Chi square tests were conducted to compare the baseline characteristics of participants between groups.

#### Seed based functional connectivity analysis

Resting state functional connectivity analysis was conducted using the CONN toolbox v18b [64] (http://www.nitrc.org/projects/conn). Preprocessing was performed using a standard pipeline in CONN. During the preprocessing, images were realigned, segmented, co-registered to each subject’s high-resolution T1 scan, and normalized to the standard Montreal Neurological Institute (MNI) template. Images were also smoothed using a 6 mm full-width at half-maximum Gaussian kernel and filtered with a frequency window of 0.008–0.09 Hz. In addition, we employed segmentation of gray matter, white matter, and cerebrospinal fluid (CSF) areas for the removal of temporal confounding factors (white matter and CSF) [64].

Data was also subjected to motion correction using the artifact detection toolbox (http://www.nitrc.org/projects/artifact_detect/). For each subject, we treated images as outliers if the composite movement from a preceding image exceeded 0.5 mm or if the global mean intensity was greater than 3 standard deviations from the mean image intensity for the entire resting scan. Outliers were included as regressors in the first-level general linear model along with six other regular motion parameters [65].

Similar to previous studies [21, 46], the bilateral medial hypothalamus (MH) seed (peak MNI coordinates: x = ± 4; y = − 2; z = − 12 plus 2 mm sphere) and lateral hypothalamus (LH) seed (peak MNI coordinates: x = ± 6; y = − 9; z = − 10 plus 2 mm sphere) were selected using WFU-Pick Atlas software. Functional connectivity measures were computed between the seed and every other voxel in the brain. First-level correlation maps were produced by extracting the residual BOLD time course from the bilateral MH and LH (respectively) and by computing Pearson's correlation coefficients between the MH or LH time courses and the time courses of all other voxels in the brain. Correlation coefficients were transformed into Fisher’s ‘Z’ scores to increase normality and allow for improved second-level general linear model analyses.

For each group (condition), a one sample t-test was applied to explore the positive and negative rsFC of pre- and post-treatment in fibromyalgia patients and HC’s respectively. Then, the baseline medial and lateral hypothalamus rsFC of fibromyalgia patients and HC’s were compared using a two-sample t-test. The Tai Chi practice effect (post-practice minus pre-practice) on fibromyalgia patients was compared using a paired t-test. For the whole brain analysis, a threshold of voxel-wise p < 0.005 (uncorrected) and cluster-level p < 0.05 (family-wise error correction) was applied for data analyses. Also, given the important role of the amygdala, rostral anterior cingulate cortex (rACC), and thalamus in the pathophysiology of fibromyalgia and pain modulation, we defined these regions as regions of interest [using the Automated Anatomical Labeling (AAL) template]. For predefined ROIs, Monte Carlo simulation using 3dFWHMx and 3dClustSim was applied, and voxel-wise p < 0.005 and p < 0.05 at cluster level were corrected for the minimum voxel. The cluster-size threshold (k) for the corrected region is shown in the results.

To explore the association between the functional connectivity association and clinical outcomes, we also performed correlation analysis between the HM/LM functional connectivity changes and the corresponding FIQR and subscores percentile changes.

### Effective connectivity of medial hypothalamus (MH)

In this study, we found that the rsFC between the MH and right thalamus decreased in fibromyalgia patients but increased after mind–body intervention (see “Results” section for details). We thus performed a spectral dynamic causal modeling (DCM) analysis using DCM 12 [66] implemented in SPM12, with the bilateral medial hypothalamus and right thalamus (i.e., overlapping areas of two contrasts: fibromyalgia vs healthy control and pre vs post Tai Chi) as regions of interest (ROIs). The following 3 models were specified: MH influencing thalamus (model 1), thalamus influencing MH (model 2), and a fully connected model of bidirectional effective connectivity between the MH and thalamus (model 3). Random effects (RFX) Bayesian Model Selection (BMS) was conducted to determine the best model for each group. For the best model, Bayesian Model Averaging (BMA) was conducted to analyze the connectivity parameters in the group level. The probability-weighted values of the model parameters were also obtained from BMA and compared across the three conditions after controlling for effects of age and gender if needed (all conditions have same best model).

## Supplementary Information

**Additional file 1: Table S1.** Individual data of demographic and clinical outcomes.

## Data Availability

Data will be available per request.
